# Cortisol, Depression, and Anxiety Levels Before and After Short-Term Intensive Nutritional Stabilization in Patients With Severe Anorexia Nervosa

**DOI:** 10.3389/fpsyt.2022.939225

**Published:** 2022-07-12

**Authors:** Simone Daugaard Hemmingsen, Nikolai Arndal Jensen, Pia Veldt Larsen, Jan Magnus Sjögren, Mia Beck Lichtenstein, René Klinkby Støving

**Affiliations:** ^1^Center for Eating Disorders, Odense University Hospital, Mental Health Services in the Region of Southern Denmark, Odense, Denmark; ^2^Research Unit for Medical Endocrinology, Department of Clinical Research, Open Patient Data Explorative Network (OPEN), University of Southern Denmark, Odense, Denmark; ^3^Mental Health Services in the Region of Southern Denmark, Vejle, Denmark; ^4^Psychiatric Center Ballerup, Copenhagen, Denmark; ^5^Department of Clinical Sciences, Umeå University, Umeå, Sweden; ^6^Centre for Telepsychiatry, Region of Southern Denmark, Vejle, Denmark; ^7^Department of Clinical Research, University of Southern Denmark, Odense, Denmark

**Keywords:** anorexia nervosa, re-nutrition, cortisol, eating disorder, depression, anxiety, weight gain, HPA axis

## Abstract

**Introduction:**

Depression and anxiety are well-known comorbid conditions in patients with anorexia nervosa (AN). Hypercortisolemia in patients with AN may be pathogenic and contribute to depression and anxiety symptomatology.

**Objective:**

The aim of this study was to investigate short-term changes in cortisol levels and depression and anxiety symptomatology following intensive re-nutrition in patients with severe AN and hospitalized in a specialized unit. Furthermore, we investigated the potential association between cortisol levels and psychometric parameters.

**Methods:**

A total of 36 patients with AN were enrolled in the study. Nine dropped out before follow-up. Patients underwent paraclinical and psychometric examinations at admission and discharge. Measurements included plasma cortisol, cortisol binding globulin (CBG), 24-h urine cortisol, and self-report questionnaires regarding eating disorder, depression, anxiety, and stress symptoms. Patients were hospitalized in the unit for somatic stabilization and intensive re-nutrition. Mean admission length was 41 days. The study was registered at ClinicalTrials.gov (NCT02502617).

**Results:**

Cortisol levels in blood and urine did not change from admission to discharge in patients with severe AN. Symptoms of depression, anxiety, stress, and eating disorder remained elevated at discharge. There were no associations between changes in cortisol levels and changes in psychometrics.

**Discussion:**

Our results suggest that short-term intensive re-nutrition did not alter hypothalamic-pituitary-adrenal axis activity or mental health in patients with severe AN. Long-term stabilization and longer follow-up after hospital discharge may be needed to detect changes in cortisol levels and whether these changes are associated with depression and anxiety symptomatology. Greater knowledge about cortisol levels and mental health in patients with severe AN may help in the development of new treatment choices for the chronically ill patients. Future studies could investigate whether cortisol-lowering drugs have a therapeutic effect on mental health in AN.

## Introduction

Anorexia nervosa (AN) is a syndrome characterized by distorted body image and fear of gaining weight. AN can be devastating to individuals and their families, and the disorder has the highest mortality rate among all psychiatric disorders (1). Most deaths in AN are related to malnutrition and accompanying somatic complications (2), but suicide is also a major cause of death in patients with AN (3). No effective evidence-based treatment is available (4, 5). The etiology is still unknown although an interplay between genetic and environmental factors has been proposed (6, 7). Genetic studies have found links to genes that may be involved in the development of the disorder (8, 9). In addition, research indicates that gut microbiome may be involved in the pathophysiology of AN (10).

Restrictive eating with accompanied malnutrition is one of the main diagnostic criteria for AN (11). A consequence of malnutrition-induced physiological stress is altered endocrine axes, leading to protein- and energy-preserving adaptation (12). Most studies investigating cortisol levels in patients with AN have found hypercortisolemia (13, 14), but it seems that cortisol levels can normalize after weight restoration in AN (14). In healthy subjects, circulating cortisol has a diurnal pattern with a stable day-night-sleep rhythm. Cortisol levels reach maximum concentrations in the morning and nadir at midnight. Cortisol circulates in the plasma in three fractions: 80% is bound to cortisol binding globulin (CBG), 10–15% is bound to albumin, and the remaining 5–10% circulates as free and biologically active cortisol (15). Estrogens stimulate CBG during treatment with oral contraception, which leads to elevated total cortisol concentrations (16). Conversely, in severe illness, the stress imposed upon the patient causes CBG to decrease (mediated by interleukin-6 levels), resulting in low total plasma cortisol levels. Correcting for CBG concentrations gives an indication of free unbound cortisol in plasma. To our knowledge, the level of CBG has not previously been assessed by new validated assays in AN.

Depression and anxiety are well-known comorbid traits in patients with AN (17), but neither anxiolytic nor antidepressant treatment has been found effective in reducing these symptoms in patients with AN (4, 18). Knowledge about the relationship between psychopathology and malnutrition in AN is currently limited to a small number of studies with contradictory conclusions (19–21). There is preliminary experimental evidence that pharmacological cortisol synthesis inhibition may have therapeutic efficacy in depression (22), but this has not yet been investigated in AN. However, one study found that cortisol levels correlated positively with anxiety and depression in patients with AN (23). Depression and anxiety are well-known symptoms in conditions with excess endogenous cortisol production (24). Elevated cortisol levels may therefore be associated with depression and anxiety symptomatology in AN.

The aim of this prospective observational study was to investigate short-term changes in cortisol (plasma cortisol, CBG, and 24-h urine cortisol) levels and mental health (depression, anxiety, stress, and eating disorder symptomatology) following intensive re-nutrition in patients with severe AN and hospitalized in a specialized unit in Denmark. Furthermore, we investigated the potential association between cortisol levels and psychometric parameters.

## Materials and Methods

The study was conducted at the Center for Eating Disorders in Odense, which is a highly specialized center with a strong, formalized collaboration between psychiatric, and somatic units. Patients with life-threatening weight loss are primarily admitted to the center’s Nutrition Unit, an inpatient unit for somatic stabilization and weight gain, after which they are either transferred to a specialized psychiatric unit or discharged to a three-armed outpatient setting combining psychiatric, somatic, and social treatment.

The median body mass index (BMI) at admission for the 84 patients in 2013 was 13.8 (7.8–25.8). The center’s organization and patient population are described in multiple observational and intervention studies (25–27). The cohort study was part of an ongoing longitudinal research project at the center that also investigates cognitive performance in severe AN.

### Recruitment

Patients diagnosed with AN in accordance with the International Classification of Diseases tenth edition (ICD-10) (28) who were hospitalized in the Nutrition Unit between 2016 and 2021 were invited to participate in the study. We included patients from across Denmark although most lived in the Region of Southern Denmark.

Patients were excluded from the study if they had active substance abuse, comorbid schizophrenia or psychosis, were under the age of 16, could not read Danish, or were admitted primarily for rapid fluid and electrolyte correction (as assessed by the chief physician). Patients were also excluded if the psychopharmacological treatment changed qualitatively during re-nutrition.

Of the 54 patients invited to participate, 36 patients agreed to participate in the study. All patients fulfilled the criteria for AN diagnosis according to the Diagnostic and Statistical Manual of Mental Disorders fifth edition (DSM-5) (11). Nine patients dropped out of the study before follow-up measurements were collected.

### Measurements

**FIGURE 1:**
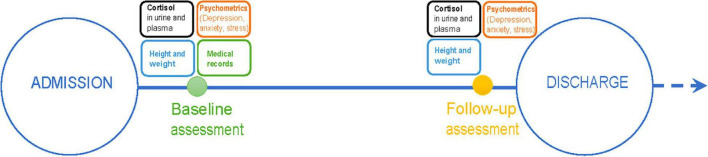
Study timeline.

Measurements consisted of clinical assessment, blood, and urine samples, and five self-report questionnaires. All measurements were conducted at admission (baseline) and at discharge (follow-up) during treatment as usual in the specialized Nutrition Unit. Admission measurements were conducted ~1 week, and no sooner than 3 days, after admission and initial stabilization of the patients. Discharge measurements were conducted a few days before discharge. In three cases, however, follow-up was conducted a few days after discharge. A timeline is presented in Figure 1.

### Clinical Measurements and Records

We measured height on a wall-mounted stadiometer and weight on a calibrated platform scale. Educational level was collected by interview. Nadir BMI, age, comorbidity, and illness duration were collected from medical records.

#### Biochemistry

All samples were stored at –80°C until analysis. Analyses were done continuously throughout the study.

Levels of cortisol and CBG were measured in venous blood samples from fasting patients between 8 and 9 a.m. Samples were prepared using a solid phase extraction method. Liquid chromatography with tandem mass spectrometry analysis was used to measure levels of cortisol in samples. Coefficient of variation was 4.4% at 560 nmol/L and 5.5% at 290 nmol/L.

Urinary cortisol levels were measured in 24-h urine samples. Samples were analyzed with liquid chromatography–mass spectrometry at Clinical Biochemistry and Pharmacology in Vejle, Denmark.

#### Psychopathology

Self-report questionnaires were sent to each participant’s e-mail address. Data were collected using Research Electronic Data Capture (REDCap) hosted at OPEN Odense Patient data Explorative Network. REDCap is a secure web platform for online databases and surveys (29, 30). The following self-assessment questionnaires were distributed.

##### Eating Disorder Symptomatology

Eating Disorder Inventory 3 (EDI-3) (31), validated in Danish (32), consists of 91 items organized into 12 subscales: Drive for Thinness (DT), Bulimia (B), Body Dissatisfaction (BD), Low Self-Esteem (LSE), Personal Alienation (PA), Interpersonal Insecurity (II), Interpersonal Alienation (IA), Interoceptive Deficits (ID), Emotional Dysregulation (ED), Perfectionism (P), Asceticism (A), and Maturity Fears (MF). It is used for clinical evaluation of symptomatology associated with eating disorders. Each question is rated on a 6-point Likert scale ranging from “always” to “never.” Afterward, answers are transformed to scores between 0 and 4. Each subscale has specific cutoff points for specific diagnostic subtypes of AN. These determine which of three groups the patient should be placed in: “Elevated clinical,” “Typical clinical,” or “Low clinical”.

##### Alexithymia

Toronto Alexithymia Scale TAS-20 (33) is a 20-item self-report instrument for assessing alexithymia (difficulty identifying and describing emotions). The questionnaire is translated into Danish by Jørgensen, Ørnbøl, and Zachariae in 2002 and the Danish translation has shown moderate to good internal consistency (34). Each question is rated on a 5-point Likert scale ranging from strongly disagree to strongly agree. The total score is the sum of all 20 items. A total score equal to or less than 51 indicates non-alexithymia, between 52 and 60 indicates intermediate alexithymia, and equal to or greater than 61 indicates alexithymia.

##### Depression, Anxiety, and Stress

Beck Depression Inventory II (BDI-II) (35) is a 21-item self-report instrument used to assess the severity of depression symptomatology. The BDI-II is widely used in clinical practice and research and is validated on a sample of participants with AN (36). It is translated into Danish (Pearson Assessment, Danish version from 2005). Each item consists of four statements. The participant chooses which statement best describes his or her feelings, thoughts, and behavior during the past 2 weeks. Answers are scored on a scale from 0 to 3, and higher scores indicate higher symptom severity. In patients diagnosed with depression, scores of 0–13 indicate minimal depression, 14–19 indicate mild depression, 20–28 indicate moderate depression, and 29–63 indicate severe depression.

Hospital Anxiety and Depression Scale (HADS) (37) is a 14-item self-report instrument, where seven items measure symptoms of anxiety and seven items measure symptoms of depression. The Danish version of the HADS is validated in patients with cardiac disease (38). Answers are rated on a 4-point Likert scale with scores from 0 to 3. For each subscale, a score of 0–7 indicates non-cases, 8–10 indicates mild symptoms, 11–14 moderate symptoms, and 15–21 severe symptoms of anxiety or depression. There is statistical evidence for the two-factor structure of the scale, and it has been validated for the assessment of anxiety and depression symptomatology in somatic and psychiatric samples as well as in the general population (39).

Perceived Stress Scale 10 (PSS-10) (40) consists of ten items assessing the degree to which people perceive their lives as stressful. The PSS-10 is validated in Danish (41). Patient responses are rated on a 5-point Likert scale ranging from “never” to “very often” and are given a score between 0 and 4. A total score ranging from 0 to 13 is considered low perceived stress, a total score of 14–26 is considered moderate perceived stress, and a total score of 27–40 is considered high perceived stress.

### Re-nutrition

Safe and effective weight restoration of 2.0–3.0% per week is the goal of the treatment in the Nutrition Unit. Individually customized meals were supervised by trained nurses at scheduled times. If the meal could not be consumed within the predefined timeframe (15 min for snacks and 30 min for main meals), supplemental nutrition drinks were added either orally or via a tube. To account for individual preferences, a choice of 3–4 different meals was offered. However, the macronutrient distribution was consistent with recommended ranges: 40–50% from carbohydrate (max 10% from sugar), 30–40% from fat, and 20–25% from protein. The daily energy intake was highly individualized according to the weight course. If a patient failed to reach 2% weekly weight gain, the energy value of the menu was increased (typically by 600 kJ). All meals were followed by a supervised rest in a seated position varying from 30 to 60 min. Between rests, light physical activity could be performed, but no forms of exercise were allowed. For patients with an excessive urge to exercise, the observation was further extended as required, up to one-to-one supervision 24 h a day. Most patients had individual agreements as to how long they were allowed outside the Nutrition Unit, ranging from 15 min to 2 h, and whether they had to be supervised or not. Nurses were allowed to search patient rooms and confiscate food, drinks, laxatives, etc. The treatment is in accordance with international guidelines (42).

### Ethics

This study followed the Declaration of Helsinki as well as the current Privacy law and the Danish Health Act regarding Patient Legal Position. The study was approved by the Regional Research Ethics Committees of Southern Denmark (project ID S-20150042) and by the Danish Data Protection Agency (registration 2008-58-0035, case number. 14/4626). The study was registered at clinicaltrials.gov prior to collection of data (NCT02502617).

Participants received an oral invitation to an informational meeting with a doctor or psychologist from the project group. Participants gave oral and written informed consent. At the meeting, participants received background information on the study and were made aware of the study purpose. It was emphasized that participation was voluntary and that they could withdraw their consent at any time without consequences. Participants were informed that a total of 50ml of blood would be drawn and that risks included small local bleeding, irritation, pain, and bruising. Participants received no benefits by participating.

### Statistics

A dropout analysis was conducted comparing participants who completed follow-up and participants who dropped out before follow-up using Wilcoxon Ranksum tests (continuous covariates) and χ^2^-tests (categorical covariates).

An approximation of free unbound cortisol in plasma was calculated by dividing plasma cortisol with CBG, which produced the variable “cortisol/CBG.”

Short-term changes in psychometric and biochemical measures before and after intensive re-nutrition were examined using a simple paired *t*-test for normally distributed variables and Wilcoxon signed rank test for non-normally distributed variables.

A sensitivity analysis was performed on 24-h urine cortisol admission vs. discharge as two participants had urine volumes below 500 ml.

Correlations between cortisol levels and psychometrics were analyzed using Spearman’s correlation (rho, ρ) for non-normally distributed variables and Pearson’s (r) for normally distributed variables on both admission/discharge measures and the change in values (i.e., delta values).

To address the risk of type 1 error due to multiple testing, we used Šidák corrected significance levels, reported for each table.

Sample size considerations were based on the FANS cohort (26, 27) as well as published literature on the EDI-3 in Danish patients with eating disorders (43), suggesting that patients with AN are expected to score 18.7 on the Drive for Thinness (DT) subscale with a standard deviation of 6.3. It was considered that a difference of 4 points on the DT subscale from admission to discharge would be clinically relevant. With 28 patients and a significance level of 5%, we could achieve a power of 90%.

All analyses were performed using STATA v. 16.1 (44) and REDCap (29).

## Results

### Baseline Characteristics

Baseline characteristics, mean length of stay, and weight gain during hospitalization for the 36 patients enrolled in the study are presented in Table 1. We calculated Cronbach’s alpha values for all the psychometric questionnaires at baseline. For all subscales α > 0.8 except for the subscales HADS-D (α = 0.77), EDI-3 PA (α = 0.76), and EDI-3 IA (α = 0.72).

**TABLE 1:** Baseline characteristics of patients with anorexia nervosa hospitalized in the Nutrition Unit.

	*n* (%)	Mean (*SD*)
Total	36 (100)	–
**Sex**		
*Female*	35 (97)	–
Age, years	–	25.3 (7.7)
Nadir body weight, kg	–	33.1 (6.9)
Nadir BMI, kg/m^2^	–	12.1 (2.1)
Baseline BMI, kg/m^2^		13.1 (2.1)
**Severity of AN according to DSM-5**		
*Mild (BMI* ≥ 17)	2 (5.6)	
*Moderate (BMI 16–16.99)*	2 (5.6)	
*Severe (BMI 15–15.99)*	2 (5.6)	
*Extreme (BMI* < *15)*	30 (83.2)	
Disease duration, years	–	7.5 (6.3)
**Diagnostic subtype**		
Restrictive	23 (64)	–
Binge/Purging	13 (36)	–
**Co-morbidity**		
None	29 (81)	–
Personality disorder, OCD, Autism spectrum	7 (19)	–
Education, years	–	12 (2.2)
Length of hospital stay, days	–	41.0 (24.5)
Weight gain during admission, kg	–	3.0 (1.8)

*SD, standard deviation, nadir: Lowest lifetime weight and/or BMI; BMI, Body Mass Index; DSM-5, Diagnostic and Statistical Manual of Mental Disorders fifth edition; OCD, Obsessive Compulsive Disorder.*

### Dropout Analysis

There were no significant differences between patients completing follow-up and dropouts on demographic characteristics, illness duration, baseline BMI, nadir BMI,% weight change during the 4 weeks prior to admission, comorbidity, or baseline psychometrics (Supplementary Table 1). One-third (33%) of the patients who dropped out had at least one comorbidity compared to 15% of the patients who completed follow-up, but this difference was not significant. All of the dropouts with comorbidity had a personality disorder.

### Biochemical Markers

Delta values (differences between admission and discharge values) were not significant for cortisol and CBG in plasma, 24-h urine cortisol, and the cortisol/CBG index representing free unbound cortisol. Results are reported in Table 2.

**TABLE 2:** Change in cortisol levels and CBG between admission and discharge.

	Admission *N* = 27		Discharge *N* = 27		
	Mean	*SD*	Mean	*SD*	*p*-value
Plasma cortisol (nmol/L)	579.7	135.1	552.8	158.2	0.41
CBG (μg/mL)	36.3	10.6	39.5	9.4	0.12
24-h urine cortisol* (nmol/L)	73.4	44.4	66.5	55.9	0.06
Cortisol/CBG	16.8	4.3	14.5	3.9	0.03

Paired t-tests and Wilcoxon signed rank test (*) were applied. Significance level (Šidák correction): α = 0.013.

*CBG, cortisol binding globulin, SD, Standard deviation.*

### Psychometrics

The patients’ responses on the psychometric questionnaires did not change significantly from admission to discharge. The EDI-3 mean subscale scores measuring eating disorder symptoms remained elevated at discharge. The BDI-II mean score measuring depression symptoms remained in the severely depressed category, the HADS mean subscale scores measuring anxiety and depression symptoms remained in the mild and moderate categories, the TAS-20 mean score measuring alexithymia remained in the category of possible alexithymia, and the PSS-10 mean score measuring perceived stress remained at the border between moderate and high perceived stress at discharge. Results of all questionnaires and subscales are reported in Table 3.

**TABLE 3:** Change in anxiety and depression scores between admission and discharge.

	Admission *N* = 27		Discharge *N* = 27	
	Mean	*SD*	Mean	*SD*	*P*-value
TAS-20*	56.5	12.0	56.5	10.8	0.81
PSS-10*	27.4	5.6	26.8	6.3	0.70
HADS anxiety*	14.2	3.7	13.3	3.6	0.22
HADS depression*	10.9	4.0	10.0	4.3	0.40
BDI-II	34.8	9.1	31.3	10.5	0.02
**EDI-3**					
DT*	20.2	7.6	19.8	6.9	0.40
B*	5.2	6.5	4.9	7.0	0.63
BD*	28.8	10.0	29.5	10.3	0.46
LSE*	16.0	5.7	16.7	5.3	0.29
PA	14.0	6.2	14.5	5.9	0.43
II	11.7	6.9	12.0	6.9	0.62
IA*	9.3	5.4	9.5	5.1	0.50
ID*	16.3	10.0	16.3	9.4	0.82
ED*	9.2	6.0	9.2	6.0	0.90
P	10.8	6.5	10.4	6.5	0.57
A*	12.1	7.2	12.5	7.4	0.76
MF	14.9	6.8	15.2	6.9	0.72

Paired t-tests and Wilcoxon signed rank tests (*) were applied. Significance level (Šidák correction): α = 0.003.

*SD, Standard deviation; TAS-20, Toronto Alexithymia Scale-20; PSS-10, Perceived Stress Scale 10; HADS, Hospital Anxiety and Depression Scale; BDI-II, Beck Depression Inventory II; EDI-3, Eating Disorder Inventory 3; DT, Drive for Thinness; B, Bulimia; BD, Body Dissatisfaction; LSE, Low Self-Esteem; PA, Personal Alienation; II, Interpersonal Insecurity; IA, Interpersonal Alienation; ID, Interoceptive Deficits; ED, Emotional Dysregulation; P, Perfectionism; A, Asceticism; MF, Maturity Fears.*

### Correlations Between Cortisol Levels and Psychometrics

None of the correlations between biochemical and psychometric variables (including all delta variables) were statistically significant. All correlations are reported in Supplementary Table 2.

## Discussion

In this prospective observational study, we found no significant reduction in cortisol levels assessed by fasting plasma concentration, CBG in plasma, 24-h urine excretion, or the free unbound cortisol index in patients with severe AN following short-term intensive stabilizing re-nutrition in an inpatient setting.

The patients were still severely depressed at discharge. In addition, anxiety, stress, and eating disorder symptomatology remained elevated at discharge.

Correlation analyses were performed on both delta values, i.e., change in biochemistry vs. change in psychometrics, and on admission/discharge cortisol measures vs. admission/discharge psychometric measures. None of the associations between biochemical variables psychometric variables were significant.

To our knowledge, this study is the first to examine the possible effects of short-term intensive re-nutrition in an inpatient setting on cortisol levels and depression, anxiety, stress, and eating disorder symptomatology as well as their interrelation in patients with severe AN.

We hypothesized that cortisol levels and depression and anxiety symptomatology would decrease following intensive re-nutrition. We also expected a relationship between change in cortisol levels and change in anxiety and depression severity. The study results did not match our expectations, however. It may still be that changes in cortisol levels are associated with changes in psychometrics in severe AN, but we would need to have a patient group who showed improvement at follow-up. There were also large differences in cortisol values (and large SD values) which may help explain the missing significant results.

A cross-sectional study by Lawson et al. found a positive relationship between pooled 12-h cortisol levels and the Hamilton rating scales for anxiety and depression in patients with AN (23). Our study was not able to confirm these results, which may be due to several reasons. Different cortisol and psychometric measurements were applied in our study, and the severity of AN was likely greater in our study as patients were hospitalized. Another study by Schmalbach et al. used a longitudinal study design and found that cortisol levels normalized following weight restoration in patients with AN (14). However, they measured salivary cortisol levels, and their sample consisted of less severely ill patients with higher BMI values than the sample in our study. In the current study, 83% of the participants were categorized as having extreme AN according to the AN severity rating in the DSM-5 (11). Our sample consisted primarily of extremely malnourished patients, the lowest with a baseline BMI value of 8.4 kg/m^2^ (previously described in a case report: (45). However, it has been argued that the DSM-5 severity ratings based on BMI levels do not reflect actual AN severity of eating disorder symptomatology (46).

Short-term intensive re-nutrition might not be sufficient to have a measurable effect on cortisol levels and psychometrics in patients with severe AN. Long-term stabilization and follow-up after hospital discharge might be needed to detect changes. However, patients admitted to the Nutrition Unit are usually critically and chronically ill. The unit receives chronic patients with severe AN, and many patients are admitted to the unit several times during their lifetime with many cases of relapse. Furthermore, low BMI values and severe malnutrition of hospitalized patients with AN warrant a high degree of caution when administering re-nutrition. Re-feeding symptoms such as edema may produce “falsely” increased weight gains shortly after admission. This might slow any weight gain as the standard increases in administered nutrition must be reduced to prevent re-feeding syndrome.

The patients in our study did significantly increase their weight, however, and electrolytes were stabilized during the intensive re-nutrition program. The primary goal of treatment was to stabilize physical health, and the patients were stabilized on varying physical and biochemical parameters, including organ functioning. The patients’ metabolism was catabolic at admission and anabolic at discharge. Despite this, cortisol levels remained unchanged at discharge, and the patients were as depressed and anxious at discharge as they were at admission. They were also still severely malnourished at discharge. Malnutrition can probably not singlehandedly explain the elevated cortisol levels found in patients with AN. Other factors such as eating disorder symptomatology, anxiety, depression, and unknown factors may influence cortisol levels. However, we could not establish an association between hypercortisolemia and psychometrics in our study.

Studies have argued that cortisol may not be an accurate way of measuring hypothalamic-pituitary-adrenal (HPA) axis activation (47). Cortisol levels might not be a suitable biological marker for severity of eating disorder, depression, and anxiety symptoms in patients with severe AN. However, it has not yet been investigated whether pharmacological cortisol synthesis inhibition has a therapeutic effect on depression and anxiety in patients with AN.

One of the strengths of our study is that it was conducted at the Nutrition Unit. Internationally, there are only a few somatic units with similar specialization and patient volume, making it suitable for studying the effects of intensive re-nutrition. All patients received similar treatment and consumed the prescribed nutrition at the same intervals. In addition, the environment was similar for all inpatients.

Based on the power calculation, the study sample size should be large enough to detect a difference of eating disorder symptomatology on the EDI-3 DT subscale, although the study would be more robust with a larger sample.

Most patients lived in the Region of Southern Denmark. However, the study retains high external validity as the sociodemographics of Funen match Denmark as a whole (48), and patients from the entire country hospitalized at the Nutrition Unit were included.

In the unit, there are two major indications for hospitalization, (1) re-nutrition during typically 4-12 weeks, or (2) solely electrolyte correction varying from 1/2 to 3 days. The latter group of patients were not included in the study. This is a potential bias. The treatment is predominantly voluntary. Compulsory treatment is only rarely applied, and if this was the case, the patient did not participate in research. Thus, the patients included in the current study were characterized by an agreement and compliance to re-nutrition and partial weight restoration (although ambivalence was present). The fluid-electrolyte correction group consists mainly of patients with the purging subtype, less severe weight loss, and/or patients who do not commit to weight gain. The disease is characterized and defined by ambivalence and aversion to any intervention that results in weight gain. This is reflected in the number of patients (*n* = 18) who did not want to participate and the number of patients (*n* = 9) who dropped out before follow-up. High dropout is well-recognized in interventions and studies of patients with AN (49). We believe this bias is a general limitation in clinical research involving patients with AN and is hard to overcome.

Participants knew the purpose of the study prior to data collection, which may be a limitation of the study. It is possible that the results were influenced by this knowledge, and the results could have been both under- and overestimated.

One questionnaire, the BDI-II, was not validated in Danish. However, it had been validated elsewhere on patients with AN, and it is widely used in clinical settings in Denmark.

Our study provides insight into the largely unknown psychopathology of AN. It is still unknown what triggers the onset of AN and whether the hyperactivation of the HPA axis is caused mainly by malnutrition or whether the onset of depression, anxiety, and eating disorder onset causes the hyperactivation, with starvation acting as a coping mechanism.

Results of our study suggest that cortisol levels and mental health in patients with severe AN are not altered by short-term intensive re-nutrition. Future avenues of treatment of AN could involve cortisol-lowering drugs and glucocorticoid receptor antagonists to investigate whether they will reduce depression and anxiety symptomatology in patients with severe AN. Fewer symptoms of depression and anxiety would increase the quality of life for patients with severe AN, even if these do not represent a cure for the eating disorder.

## Data Availability Statement

The datasets presented in this article are not readily available because individuals may be identified. Currently, we only have permission to store the datasets in places where access and modifications are logged. Requests to access the datasets should be directed to corresponding author.

## Ethics Statement

The studies involving human participants were reviewed and approved by the Regional Research Ethics Committees of Southern Denmark. Written informed consent to participate in this study was provided by the participants’ legal guardian/next of kin.

## Author Contributions

RS was the initiator of the research project. SH and RS developed the database and completed the data collection. SH, RS, and ML took part in the design of the study and wrote the protocol. NJ, PL, and SH carried out the statistics. SH, NJ, RS, PL, ML, and JS were all contributors in writing the manuscript. SH, NJ, and RS contributed to all parts of the manuscript. PL primarily contributed to the result section, and sections of the abstract, the method, and the discussion related to results. ML and JS primarily contributed to the introduction and discussion sections. All authors read and approved the final manuscript.

## Conflict of Interest

The authors declare that the research was conducted in the absence of any commercial or financial relationships that could be construed as a potential conflict of interest.

## Publisher’s Note

All claims expressed in this article are solely those of the authors and do not necessarily represent those of their affiliated organizations, or those of the publisher, the editors and the reviewers. Any product that may be evaluated in this article, or claim that may be made by its manufacturer, is not guaranteed or endorsed by the publisher.
