# Supplementary material for: Cortisol, Depression, and Anxiety Levels Before and After Short-Term Intensive Nutritional Stabilization in Patients With Severe Anorexia Nervosa
Source: Front Psychiatry. 2022 Jul 12;13:939225. doi: 10.3389/fpsyt.2022.939225 (PMC9314772; doi:10.3389/fpsyt.2022.939225)
Supplement: Supplementary file 1 [file Table_1.pdf]

Supplementary Material

1. Dropout analysis

Supplementary Table 1. Demographic and baseline characteristics of participants who completed follow-up measurements and participants who dropped out of the study

	**Completers**	**Dropouts**	***p*-value**
**Total, *n***	27	9	
**Age, years**<br>**Median (IQR)**	22 (7)	27 (9)	0.080
**Illness duration, years**<br>**Median (IQR)**	4 (6)	10 (10)	0.090
**Comorbidity, *n* (%)**	4 (15)	3 (33)	0.224
**Nadir BMI**<br>**Median (IQR)**	12.1 (2.2)	11.3 (3.8)	0.780
**Education, years**<br>**Median (IQR)**	12 (3)	12 (3)	0.371
**BMI baseline**<br>**Median (IQR)**	12.9 (2.4)	12.7 (5.2)	0.666
**4 weeks weight change, %**<br>**Median (IQR)**	-3.9 (8.7)	0 (8.8)	0.192
**TAS-20 total score**<br>**Median (IQR)**	58 (21)	58 (17)	0.511
**PSS-10 total score**<br>**Median (IQR)**	28 (8)	26 (3)	0.712
**HADS anxiety subscale**<br>**Median (IQR)**	14 (4)	12 (6)	0.206
**HADS depression subscale**<br>**Median (IQR)**	11 (4)	7 (6)	0.065
**BDI-II totalscore**<br>**Median (IQR)**	36 (14)	27 (15)	0.274
**EDI-3 DT subscale**<br>**Median (IQR)**	22 (9)	16 (16)	0.265
**EDI-3 B subscale**<br>**Median (IQR)**	3 (9)	7 (9)	0.198
**EDI-3 BD subscale**<br>**Median (IQR)**	32 (15)	28 (22)	0.534

**EDI-3 LSE subscale**			
**Median (IQR)**	18 (6)	13 (10)	0.169
**EDI-3 PA subscale**			
**Median (IQR)**	13 (8)	13 (7)	0.950
**EDI-3 II subscale**			
**Median (IQR)**	12 (9)	7 (13)	0.646
**EDI-3 IA subscale**			
**Median (IQR)**	7 (9)	6 (11)	0.522
**EDI-3 ID subscale**			
**Median (IQR)**	17 (14)	17 (14)	0.921
**EDI-3 ED subscale**			
**Median (IQR)**	10 (11)	11 (15)	>0.999
**EDI-3 P subscale**			
**Median (IQR)**	9 (12)	13 (7)	0.978
**EDI-3 A subscale**			
**Median (IQR)**	14 (14)	10 (9)	0.907
**EDI-3 MF subscale**			
**Median (IQR)**	14 (12)	8 (10)	0.195

n=3 dropouts did not complete the questionnaires but completed urine and blood samples at follow-up.

IQR: interquartile range, BMI: body mass index, TAS-20: Toronto Alexithymia Scale-20, PSS-10: Perceived Stress Scale 10, HADS: Hospital Anxiety and Depression Scale, BDI-II: Beck Depression Inventory II, EDI-3: Eating Disorder Inventory 3, DT: Drive for Thinness, B: Bulimia, BD: Body Dissatisfaction, LSE: Low Self-Esteem, PA: Personal Alienation, II: Interpersonal Insecurity, IA: Interpersonal Alienation, ID: Interoceptive Deficits, ED: Emotional Dysregulation, P: Perfectionism, A: Asceticism, and MF: Maturity Fears.

2. Correlations between Cortisol levels and Psychometrics

The following table reports all correlations between cortisol and psychometric variables including delta variables.

Supplementary Table 2. Correlations between biochemical and psychometric variables

	vs.	rho	*p*-value
Delta Cortisol/CBG	Delta TAS_20	0.03	0.895
	Delta PSS-10	0.08	0.719
	Delta HADS-A	-0.04	0.855
	Delta HADS-D	-0.18	0.400
	Delta BDI-II	-0.18	0.396
	Delta EDI-3 DT	0.05	0.816
	Delta EDI-3 B	-0.05	0.799
	Delta EDI-3 BD	-0.11	0.590
	Delta EDI-3 LSE	0.03	0.878
	Delta EDI-3 PA	0.06	0.773
	Delta EDI-3 II	0.07	0.743

	Delta EDI-3 IA	0.13	0.522
	Delta EDI-3 ID	0.20	0.332
	Delta EDI-3 ED	-0.16	0.438
	Delta EDI-3 P	0.17	0.405
	Delta EDI-3 A	0.17	0.416
	Delta EDI-3 MF	0.13	0.538
Delta 24-Hour Urine Cortisol	Delta TAS_20	0.27	0.188
	Delta PSS-10	-0.19	0.361
	Delta HADS-A	-0.07	0.744
	Delta HADS-D	-0.03	0.883
	Delta BDI-II	0.12	0.575
	Delta EDI-3 DT	0.08	0.704
	Delta EDI-3 B	0.17	0.413
	Delta EDI-3 BD	-0.05	0.805
	Delta EDI-3 LSE	-0.04	0.866
	Delta EDI-3 PA	0.13	0.538
	Delta EDI-3 II	0.26	0.201
	Delta EDI-3 IA	-0.21	0.316
	Delta EDI-3 ID	-0.18	0.395
	Delta EDI-3 ED	0.38	0.061
	Delta EDI-3 P	0.43	0.031
	Delta EDI-3 A	0.35	0.088
	Delta EDI-3 MF	-0.20	0.330
Delta plasma cortisol	Delta TAS_20	-0.22	0.300
	Delta PSS-10	-0.13	0.528
	Delta HADS-A	0.22	0.291
	Delta HADS-D	-0.16	0.447
	Delta BDI-II	-0.20	0.326
	Delta EDI-3 DT	-0.04	0.859
	Delta EDI-3 B	-0.34	0.094
	Delta EDI-3 BD	-0.09	0.667
	Delta EDI-3 LSE	-0.08	0.699
	Delta EDI-3 PA	0.06	0.782
	Delta EDI-3 II	0.10	0.638
	Delta EDI-3 IA	0.07	0.746
	Delta EDI-3 ID	0.07	0.740
	Delta EDI-3 ED	-0.12	0.569
	Delta EDI-3 P	0.19	0.351
	Delta EDI-3 A	-0.13	0.526
	Delta EDI-3 MF	0.32	0.120
Baseline plasma cortisol	Baseline TAS_20	-0.19	0.338
	Baseline PSS-10	0.09	0.655
	Baseline HADS-A	0.20	0.328
	Baseline HADS-D	-0.14	0.476
	Baseline BDI-II	-0.13	0.513
	Baseline EDI-3 DT	0.08	0.694
	Baseline EDI-3 B	0.28	0.163
	Baseline EDI-3 BD	-0.11	0.585
	Baseline EDI-3 LSE	-0.18	0.376

	Baseline EDI-3 PA	-0.09	0.670
	Baseline EDI-3 II	-0.24	0.248
	Baseline EDI-3 IA	-0.14	0.472
	Baseline EDI-3 ID	0.06	0.769
	Baseline EDI-3 ED	-0.18	0.361
	Baseline EDI-3 P	-0.05	0.817
	Baseline EDI-3 A	-0.20	0.314
	Baseline EDI-3 MF	0.19	0.339
Baseline 24-Hour Urine Cortisol	Baseline TAS_20	-0.12	0.551
	Baseline PSS-10	-0.31	0.120
	Baseline HADS-A	-0.30	0.130
	Baseline HADS-D	-0.28	0.157
	Baseline BDI-II	-0.39	0.042
	Baseline EDI-3 DT	-0.55	0.003
	Baseline EDI-3 B	-0.17	0.385
	Baseline EDI-3 BD	-0.46	0.017
	Baseline EDI-3 LSE	-0.36	0.065
	Baseline EDI-3 PA	-0.26	0.187
	Baseline EDI-3 II	-0.11	0.600
	Baseline EDI-3 IA	-0.31	0.112
	Baseline EDI-3 ID	-0.18	0.382
	Baseline EDI-3 ED	-0.32	0.101
	Baseline EDI-3 P	-0.41	0.035
	Baseline EDI-3 A	-0.25	0.207
	Baseline EDI-3 MF	0.19	0.339
Baseline Cortisol/CBG	Baseline TAS_20	0.01	0.953
	Baseline PSS-10	0.05	0.800
	Baseline HADS-A	-0.03	0.871
	Baseline HADS-D	0.22	0.274
	Baseline BDI-II	-0.07	0.725
	Baseline EDI-3 DT	-0.15	0.454
	Baseline EDI-3 B	0.30	0.127
	Baseline EDI-3 BD	-0.51	0.007
	Baseline EDI-3 LSE	-0.14	0.488
	Baseline EDI-3 PA	-0.11	0.591
	Baseline EDI-3 II	-0.02	0.937
	Baseline EDI-3 IA	-0.05	0.789
	Baseline EDI-3 ID	-0.05	0.823
	Baseline EDI-3 ED	-0.26	0.196
	Baseline EDI-3 P	-0.40	0.039
	Baseline EDI-3 A	-0.11	0.572
	Baseline EDI-3 MF	0.48	0.011
Follow-up plasma cortisol	Follow-up TAS_20	0.06	0.778
	Follow-up PSS-10	-0.08	0.711
	Follow-up HADS-A	-0.01	0.956
	Follow-up HADS-D	-0.11	0.597
	Follow-up BDI-II	0.01	0.956
	Follow-up EDI-3 DT	-0.33	0.102

	Follow-up EDI-3 B	-0.13	0.540
	Follow-up EDI-3 BD	-0.29	0.160
	Follow-up EDI-3 LSE	-0.38	0.059
	Follow-up EDI-3 PA	-0.05	0.830
	Follow-up EDI-3 II	0.26	0.218
	Follow-up EDI-3 IA	-0.29	0.162
	Follow-up EDI-3 ID	0.01	0.968
	Follow-up EDI-3 ED	-0.08	0.721
	Follow-up EDI-3 P	-0.17	0.407
	Follow-up EDI-3 A	0.06	0.770
	Follow-up EDI-3 MF	0.08	0.700
Follow-up 24-Hour Urine Cortisol	Follow-up TAS_20	-0.03	0.874
	Follow-up PSS-10	-0.21	0.324
	Follow-up HADS-A	-0.11	0.593
	Follow-up HADS-D	-0.11	0.587
	Follow-up BDI-II	-0.12	0.564
	Follow-up EDI-3 DT	-0.38	0.059
	Follow-up EDI-3 B	-0.18	0.397
	Follow-up EDI-3 BD	-0.46	0.022
	Follow-up EDI-3 LSE	-0.35	0.083
	Follow-up EDI-3 PA	-0.03	0.875
	Follow-up EDI-3 II	0.16	0.457
	Follow-up EDI-3 IA	-0.20	0.350
	Follow-up EDI-3 ID	-0.17	0.420
	Follow-up EDI-3 ED	-0.17	0.422
	Follow-up EDI-3 P	-0.43	0.034
	Follow-up EDI-3 A	-0.15	0.482
	Follow-up EDI-3 MF	-0.01	0.950
Follow-up Cortisol/CBG	Follow-up TAS_20	0.11	0.615
	Follow-up PSS-10	-0.10	0.636
	Follow-up HADS-A	-0.20	0.343
	Follow-up HADS-D	-0.28	0.174
	Follow-up BDI-II	-0.28	0.169
	Follow-up EDI-3 DT	-0.25	0.219
	Follow-up EDI-3 B	-0.20	0.343
	Follow-up EDI-3 BD	-0.18	0.384
	Follow-up EDI-3 LSE	-0.39	0.054
	Follow-up EDI-3 PA	-0.19	0.365
	Follow-up EDI-3 II	-0.21	0.320
	Follow-up EDI-3 IA	-0.41	0.040
	Follow-up EDI-3 ID	-0.08	0.721
	Follow-up EDI-3 ED	-0.22	0.302
	Follow-up EDI-3 P	-0.20	0.331
	Follow-up EDI-3 A	0.14	0.517
	Follow-up EDI-3 MF	-0.06	0.779

Analysis was performed using Spearman’s correlation. **Significance level (Šidák correction): $\alpha = 3.351941 \times 10^{-4}$**.

CBG: cortisol binding globulin, TAS-20: Toronto Alexithymia Scale-20, PSS-10: Perceived Stress Scale 10, HADS: Hospital Anxiety and Depression Scale, BDI-II: Beck Depression Inventory II, EDI-3: Eating Disorder Inventory 3, DT: Drive for Thinness, B: Bulimia, BD: Body Dissatisfaction, LSE: Low Self-Esteem, PA: Personal Alienation, II: Interpersonal Insecurity,
IA: Interpersonal Alienation, ID: Interoceptive Deficits, ED: Emotional Dysregulation,
P: Perfectionism, A: Asceticism, and MF: Maturity Fears.
